# A Retrospective Cohort Study of Disparities in Urine Drug Testing During the Perinatal Period in an Urban, Academic Medical Center

**DOI:** 10.1007/s10995-024-03940-4

**Published:** 2024-06-07

**Authors:** Valerie S. Ganetsky, Brianna Yates, Matthew Salzman, Jessica Heil, Iris Jones, Krystal Hunter, Robin L. Perry, Kaitlan E. Baston

**Affiliations:** 1 Center for Healing, Division of Addiction Medicine, Cooper University Health Care, Camden, NJ USA; 2 Department of Family and Community Medicine, Thomas Jefferson University, Philadelphia, PA USA; 3 Department of Emergency Medicine, Division of Addiction Medicine and Medical Toxicology, Cooper University Health Care, Camden, NJ USA; 4 Cooper University Health Care, Cooper Research Institute, Camden, NJ USA; 5 Department of Obstetrics and Gynecology, Cooper University Health Care, Camden, NJ USA

**Keywords:** Substance use, Health disparities, Prenatal care, Urine drug testing

## Abstract

The purpose of this study was to evaluate disparities in urine drug testing (UDT) during perinatal care at a single academic medical center. This retrospective cohort study included patients who had a live birth and received prenatal care at our institution between 10/1/2015 and 9/30/2020. The primary outcomes were maternal UDT during pregnancy (UDTPN) and UDT only at delivery (UDTDEL). Secondary outcomes included the number of UDTs (UDTNUM) and the association between a positive UDT test result and race/ethnicity. Mixed model logistic regression and negative binomial regression with clustering based on prenatal care locations were used to control for confounders. Of 6,240 live births, 2,265 (36.3%) and 167 (2.7%) received UDTPN and UDTDEL, respectively. Black (OR 2.09, 95% CI 1.54–2.84) and individuals of Other races (OR 1.64, 95% CI 1.03–2.64) had greater odds of UDTPN compared to non-Hispanic White individuals. Black (beta = 1.12, *p* < 0.001) and Hispanic individuals (beta = 0.78, *p* < 0.001) also had a positive relationship with UDTNUM. Compared to individuals with non-Medicaid insurance, those insured by Medicaid had greater odds of UDTPN (OR 1.66, 95% CI 1.11–2.49) and had a positive relationship with UDTNUM (beta = 0.89, *p* < 0.001). No significant associations were found for UDTDEL and race/ethnicity. Despite receiving more UDT, Black individuals were not more likely to have a positive test result compared to non-Hispanic White individuals (OR 0.95, 95% CI 0.72–1.25). Our findings demonstrate persistent disparities in substance use testing during the perinatal period.

## Significance

**What is already known on this subject?:**

Prior literature has shown evidence of racial disparities in urine drug testing during pregnancy-related hospital admissions and at delivery.

**What this study adds?:**

This paper found that disparities in urine drug testing also exist during the prenatal care period at an urban academic institution. We also found a positive relationship between the number of tests received and being of Black or Hispanic race/ethnicity. Future research should investigate health care system-related interventions to mitigate disparities in urine drug testing.

## Introduction

Substance use during pregnancy is common. Recent estimates indicate that nearly 10% of pregnant individuals used illicit drugs in the past month (SAMHSA, 2022). Paralleling the U.S. opioid epidemic, rates of maternal opioid-related diagnoses have also increased over time by 131% between 2010 and 2017 (Hirai et al., 2021). Pregnant individuals who use substances carry an increased risk of adverse perinatal outcomes, including preterm birth and miscarriage (Wright et al., 2016; Viteri et al., 2015). As such, pregnancy presents a critical window of opportunity for substance use screening and intervention (Jones et al., 2014; Wright et al., 2016).

The American College of Obstetricians and Gynecologists recommends universal verbal substance use screening at the initial prenatal care visit using a validated screening tool (e.g., 4Ps, National Institute on Drug Abuse “NIDA” Quick Screen) for all pregnant patients, irrespective of race, ethnicity, or socioeconomic status (SES) (Ecker et al., 2019; ACOG, 2017). Nonetheless, biologic testing via urine drug testing (UDT) is still commonly practiced in lieu of verbal screening among obstetric providers (Wright et al., 2016). However, UDT is unable to characterize the frequency and severity of substance use or diagnose use disorders and is fraught with false-positive and false-negative results. Furthermore, UDT is often done selectively and without documentation of informed consent by the patient (Terplan & Minkoff, 2017; Winchester et al., 2022). A positive result on a UDT can have devastating consequences for families, including stigmatization and child welfare involvement leading to family separation. Literature has consistently found racial disparities in receipt of UDT, thus contributing to biased and inappropriate reporting to child welfare agencies (Chasnoff et al., 1990; Jones et al., 2014; Roberts et al., 2015; Terplan & Minkoff, 2017; Wright et al., 2016).

Studies published over a decade ago found evidence of racial disparities in perinatal UDT (Kerker et al., 2004; Kunins et al., 2007). Racial disparities persisted in more recent studies that evaluated UDT during inpatient obstetric admissions (Chin et al., 2022; Jarlenski et al., 2023; Perlman et al., 2022; Winchester et al., 2022). Currently available literature evaluated disparities in testing during hospital admissions. Our study was designed to fill an evidence gap on disparities in UDT during prenatal care. The aim of this study was to conduct an evaluation of disparities during the prenatal period and add to the current literature on disparities at delivery to examine whether race continues to be a factor in receipt of UDT. We hypothesized that Black individuals and those of lower SES would be more likely than non-Hispanic White individuals and those of higher SES to receive UDT despite controlling for potential confounders.

## Methods

### Data Sources and Collection

In this retrospective cohort study, all study variables were extracted electronically from the electronic medical record (EMR) of a large, academic health system. The Institutional Review Board approved this study.

### Study Population

Individuals who had a live birth at our institution and received prenatal care at one of the institution’s outpatient obstetrics/gynecology (OB/GYN) practices between 10/1/2015 and 9/30/2020 were included in the study. Each live birth was considered its own treatment episode. We also conducted a sensitivity analysis that only included the first live birth for any individuals with more than one live birth during the study period. Additionally, in order to avoid analysis of duplicated data, we included only unique encounters for those with a multiple gestation pregnancy. No formal policy for UDT during pregnancy existed at our institution during the study period.

### Outcome Variables

The primary outcome consisted of two separate analyses: maternal receipt of UDT at any time during pregnancy (UDTPN) and maternal receipt of UDT at delivery only (UDTDEL). These variables were dichotomous and coded as ‘yes’ if the individual had UDT (regardless of the test result). The secondary outcome was total number of UDTs performed during pregnancy (UDTNUM) and the association between a positive UDT test result and race/ethnicity. 

### Factors Examined

The following variables were observed in our study in either the bivariate and/or multivariate analysis: self-reported race/ethnicity (categorized as non-Hispanic White, Black, Hispanic, Asian or Pacific Islander and Other (includes those that self-identified as Native American-Alaskan, Multi-racial, those marked as “Other”, and those marked as “Declined to Answer”), age, insurance type (based on payor at the delivery encounter), employment status, marital status, prenatal care-related factors [gestational age at first prenatal care visit, number of prenatal care visits during pregnancy, location of prenatal care (designated as the location where the majority of prenatal care visits took place)], obstetric history at the delivery encounter (gravidity; parity; number of term births, preterm births, and abortions), maternal comorbidities [presence of mental health condition(s), human immunodeficiency virus (HIV), sexually transmitted infections (STIs), and hepatitis C virus diagnosis present prior to delivery based on International Classification of Disease, Tenth Revision (ICD-10) codes], and substance use history [categorized as either presence of an SUD based on ICD-10 codes or documentation of substance use]. Substance use (including tobacco, alcohol, and non-prescribed substances) was based on documentation in the EMR’s social history. SUD and substance use were further categorized as ‘during pregnancy’ (within 280 days of delivery) or ‘past history’ (anytime prior to delivery).

For the analyses of UDT at delivery we added obstetric diagnoses (preeclampsia, eclampsia, placenta previa, preterm labor, intrauterine growth restriction, third-trimester bleeding, and placental abruption) and newborn-related variables (birth weight as a continuous variable).

### Statistical Analysis

We used a full population cohort of patients who met the inclusion criteria during the 5-year study timeframe. No sample size calculation was performed. Descriptive statistics reporting means (with standard deviation), medians (with interquartile range), and frequencies (with percents) were used to describe baseline patient characteristics. With the exception of the history of preterm births and abortions, fewer than 3% of variables had missing data. Any missing data was handled using listwise deletion.

Initial bivariable analyses were conducted to compare all independent variables between those patients who received UDTPN and UDTDEL versus those who did not. We used independent t-tests or Mann-Whitney U tests for continuous variables and Chi-square tests for race/ethnicity and other categorical variables. Mixed model logistic and negative binomial regression models were used to evaluate factors associated with study outcomes. Mixed model regressions were performed for UDTPN and UDTDEL. Negative binomial regression analyses were performed for UDTNUM. All models included the following covariates: age, gestational age at first prenatal care visit, payor, mental health status, previous history of SUD, and parity. Covariate selection was determined using backward elimination. Insurance type was categorized as Medicaid versus non-Medicaid. We also created a variable for STIs where we composited the variables for STIs, hepatitis C virus, and HIV into one STI variable. We also created interaction terms for race x Medicaid coverage for UDTPN and UDTDEL and race x previous SUD for UDTPN only as there was not enough data to evaluate UDTDEL . We included a cluster analysis where the data was clustered by prenatal care location in order to account for differences among locations. We also conducted a multivariable logistic regression model to evaluate the association between a positive UDT result and race/ethnicity, adjusting for the covariates above.

For the primary outcomes, we validated the models by making an 80/20 division of data. 80% of the data comprised our training data while 20% of the data was used for testing. We validated data by comparing the percent correct prediction and the area under the curve value for the main test and training data. The main model that had full data had a percent correct prediction estimation of 79.3% and an area under the curve (AUC) of 0.85 (95% CI 0.84–0.86). The training data for UDTPN had the same AUC with a percent correct prediction estimation of 79.5% and testing data had a percent correct prediction estimation of 78.8% and a similar AUC, indicating that there is limited to non-existent overfitting of the data. Validation of UDTDEL data found that the unsplit, training, and testing data had percent correct prediction estimations of 93.5%, 93.4%, and 93.9%, respectively. The AUCs for these were 0.72 (95% CI 0.69–0.76), 0.72 (95% CI 0.68–0.77), and 0.80 (95% CI 0.73–0.87), respectively.

We conducted several sensitivity analyses. One sensitivity analysis removed data from 2020 for UDTPN and UDTDEL to account for the COVID-19 pandemic. A second sensitivity analysis included only the first live birth for any individual with more than one live birth during the study period. Statistical analyses were performed using SPSS 27 (IBM, Armon, NY). A p value of < 0.05 was considered statistically significant.

## Results

The study included 6,410 live births of individuals who received their prenatal care at one of our institution’s outpatient OB/GYN practices from October 2015 to September 2020, representing 6,240 unique births (after accounting for multiple gestation pregnancies). Non-Hispanic White individuals accounted for 41.5% of the total study population, while 28.0% identified as Black. Nearly half (49.6%) of individuals were insured by Medicaid and the average gestational age at the first prenatal care visit was 16 weeks. Of 6,240 live births, 2,265 (36.3%) and 167 (2.7%) received UDTPN and UDTDEL, respectively.Of all UDTs performed (n=2,432), 6.9%were at the delivery encounter. Multiple UDTs were completed in 875 patients (14.0%). Tables 1 and 2 display the baseline characteristics based on receipt of UDTPN or UDTDEL.

**Table 1:** Sociodemographic and clinical characteristics by receipt of UDT during prenatal care (*N* = 6240)

Characteristic	UDT = No		UDT = Yes		*P* value
	N^†^	n (%)	N^‡†^	n (%)	
Age, mean ± SD	3975	30.1 ± 5.69	2265	27.6 ± 6.1	< 0.001
*Race/ethnicity (self-reported)*	3975		2265		< 0.001
Non-Hispanic White		2017 (50.7)		566 (25.0)	
Black		824 (20.7)		918 (40.5)	
Hispanic		611 (15.4)		625 (27.6)	
Asian/Pacific Islander		277 (7.0)		60 (2.6)	
Other^§^		246 (6.2)		96 (4.2)	
*Insurance payor*	3975		2265		< 0.001
Medicaid		1417 (35.6)		1696 (74.9)	
Non-Medicaid^||^		2558 (64.4)		569 (25.1)	
*Marital status*	3974		2265		< 0.001
Single		1859 (46.8)		1840 (81.2)	
Married/domestic partner		1995 (50.2)		377 (16.6)	
Divorced/separated/widowed		120 (3.0)		48 (2.1)	
*Employment status*	3905		2217		< 0.001
Employed		2348 (60.1)		947(42.7)	
*Prenatal care*	3975		2265		
Gestational age at first visit, mean ± SD		16.2 ± 7.6		15.7 ± 8.3	< 0.001
Number of prenatal care visits, mean ± SD		10.9 (3.8)		9.5 (4.4)	< 0.001
Location of prenatal care					< 0.001
Inner-city		799 (20.1)		1712(75.6)	
Non-inner-city		3176 (79.9)		553(24.4)	
*Obstetric history, median (range)*					
Gravidity	3975	3 (1–15)	2265	3 (1–16)	< 0.001
Parity	3975	2 (0–10)	2264	2 (1–11)	< 0.001
Term births	3888	2 (0–9)	2185	2 (0–10)	< 0.001
Preterm births	1783	0 (0–5)	1405	0 (0–7)	< 0.001
Abortions	2493	1 (0–13)	1682	1 (0–14)	< 0.001
Maternal comorbidities	3975		2265		
Mental health diagnosis		1154 (29.0)		904 (39.9)	< 0.001
Sexually transmitted infection		186 (4.7)		268 (11.8)	< 0.001
Hepatitis C virus		19 (0.5)		68 (3.0)	< 0.001
HIV		16 (0.4)		21 (0.9)	0.009
Maternal substance use history	3975		2265		
SUD (during pregnancy)		77 (1.9)		331 (14.6)	< 0.001
SUD (past history)		114 (2.9)		386 (17.0)	< 0.001
Substance use (during pregnancy)††		213 (5.4)		493 (21.8)	< 0.001
Substance use (past history)††		336 (8.5)		667 (29.4)	< 0.001
Tobacco (during pregnancy)		361 (9.1)		601 (26.5)	< 0.001
Alcohol (during pregnancy)		1225 (30.8)		531 (23.4)	< 0.001

UDT, urine drug testing; SD, standard deviation; HIV, human immunodeficiency virus; SUD, substance use disorder

^†^Total number of patients for whom UDT was not performed taking into account missing data

^‡^Total number of patients for whom UDT was performed taking into account missing data

^§^Includes those that self-identified as Native American-Alaskan, multi-racial, those marked as “Other”, and those marked as “Declined to Answer”

^||^Non-Medicaid included commercial or military insurance, self-pay, or unknown insurance payer

^†^Substance use is based on whether an individual was marked as someone who uses “illicit drugs” in the social history portion of the medical chart t

**Table 2:** Sociodemographic and clinical characteristics by receipt of UDT at delivery only (*N* = 2506)

Characteristic	UDT = No		UDT = Yes		*P* value
	N†	n (%)	N‡	n (%)	
Age, mean ± SD	2339	27.5 ± 6.0	167	28.5 ± 6.1	0.044
*Race/ethnicity (self-reported)*	2339		167		0.005
Non-Hispanic White		611 (26.1)		65 (38.9)	
Black		928 (39.7)		55 (32.9)	
Hispanic		634 (27.1)		33 (19.8)	
Asian/Pacific Islander		63 (2.7)		609 (2.6)	
Other§		103 (4.4)		9 (5.4)	
*Insurance payor*	2339		167		0.016
Medicaid		1726 (73.8)		109 (65.3)	
Non-Medicaid||		613 (26.2)		58 (34.7)	
*Marital status*	2339		167		0.065
Single		1885 (80.6)		125 (74.9)	
Married/domestic partner		400 (17.1)		34 (20.4)	
Divorced/separated/widowed		54 (2.3)		8 (4.8)	
*Employment status*	2286		159		0.144
Employed		969 (42.4)		58 (36.5)	
*Prenatal care*	2339		167		
Gestational age at first visit, mean ± SD		16.0 ± 8.5		20.9 ± 9.0	< 0.001
Number of prenatal care visits, mean ± SD		9.4 (4.5)		6.2 (3.8)	< 0.001
Location of prenatal care	2339		167		< 0.001
Inner-city		1730 (74.0)		98 (58.7)	
Non-inner-city		609 (26.0)		69 (41.3)	
*Obstetric history, median (range)*					
Gravidity	2339	3 (1–16)	167	3 (1–14)	0.711
Parity	2338	2 (1–11)	167	2 (0–8)	0.748
Term births	2247	2 (0–10)	147	2 (0–8)	0.602
Preterm births	1457	0 (0–7)	108	1 (0–4)	0.004
Abortions	1735	1 (0–14)	117	1 (0–8)	0.130
*Obstetric diagnoses*	2339		167		
Preeclampsia		130 (5.6)		4 (2.4)	0.079
Placenta previa		89 (3.8)		2 (1.2)	0.083
Preterm labor		123 (5.3)		18 (10.8)	0.003
Intrauterine growth restriction		74 (3.2)		5 (3.0)	0.903
Third-trimester bleeding		80 (3.4)		3 (1.8)	0.257
Eclampsia		4 (0.2)		1 (0.6)	0.292
Placental abruption		5 (0.2)		1 (0.6)	0.339
*Maternal comorbidities*	2339		167		
Mental health diagnosis		930 (39.8)		72 (43.1)	0.393
Sexually transmitted infection		262 (11.2)		13 (7.8)	0.172
Hepatitis C virus		38 (1.6)		68 (40.7)	0.342
HIV		22 (0.9)		3 (1.8)	0.230
*Maternal substance use history*	2339		167		
SUD (during pregnancy)		347 (14.8)		35 (21.0)	0.033
SUD (past history)		408 (17.4)		43 (25.7)	0.007
Substance use (during pregnancy)††		514 (22.0)		38 (22.8)	0.814
Substance use (past history)††		685 (29.3)		55 (32.9)	0.318
Tobacco (during pregnancy)		619 (26.5)		66 (39.5)	< 0.001
Alcohol (during pregnancy)		540 (23.1)		36 (21.6)	0.650

UDT, urine drug testing; SD, standard deviation; HIV, human immunodeficiency virus; SUD, substance use disorder

†Total number of patients for whom UDT was not performed taking into account missing data

‡Total number of patients for whom UDT was performed taking into account missing data

§Includes those that self-identified as Native American-Alaskan, multi-racial, those marked as “Other”, and those marked as “Declined to Answer”

||Non-Medicaid included commercial or military insurance, self-pay, or unknown insurance payer

††Substance use is based on whether an individual was marked as someone who uses “illicit drugs” in the social history portion of the medical chart

### Bivariable Analyses

Compared to non-Hispanic White individuals, the unadjusted bivariable analysis of UDTPN found statistically significant associations among Black (OR 3.97, 95% CI 3.48–4.53), Hispanic (OR 3.65, 95% CI 3.15–4.22), and individuals of Other races (OR 1.39, 95% CI 1.08–1.79). No statistically significant differences were found in unadjusted analyses for UDTDEL (results not included in tables).

### Multivariable Regression Analyses

Table 3 shows the results of the multivariable logistic regression model of factors associated with receipt of UDTPN or at UDTDEL and Table 4 shows the results of the negative binomial regression model of factors associated with UDTNUM.

**Table 3:** Multivariable logistic regression model of factors associated with receipt of UDT during pregnancy (UDTPN) or at delivery (UDTDEL)

Variable	UDTPN OR (95% CI)	UDTDEL OR (95% CI)
Age	**0.98 (0.96–0.99)**	1.00 (0.97–1.04)
*Race/ethnicity*		
Non-Hispanic White	Ref	Ref
Black	**2.09 (1.54–2.84)**	0.90 (0.45–1.82)
Hispanic	1.31 (0.92–1.85)	0.98 (0.41–2.33)
Asian/Pacific Islander	1.04 (0.62–1.74)	1.97 (0.60–6.45)
Other	**1.64 (1.03–2.64)**	0.42 (0.09–1.95)
*Insurance type*		
Non-Medicaid	Ref	Ref
Medicaid	**1.27 (1.04–1.54)**	0.98 (0.54–1.78)
*Prenatal care*		
Gestational age at first visit	**0.96 (0.95–0.98)**	**1.06 (1.04–1.07)**
*Obstetric history*		
Parity	**1.07 (1.02–1.14)**	1.05 (0.93–1.20)
*Maternal comorbidities*		
Mental health diagnosis		
No	Ref	
Yes	**1.35 (1.16–1.58)**	0.96 (0.67–1.27)
STI, HIV, Hepatitis C		
No	Ref	
Yes	**1.41 (1.13–1.75)**	0.96 (0.58–1.60)
*Maternal substance use history*		
SUD (past history)		
No	Ref	
Yes	**4.17 (2.77–6.27)**	**1.48 (1.03–2.15)**
Newborn birth weight (ounces)	-	**0.99 (0.98–0.99)**
Black race x Medicaid	**0.57 (0.40–0.82)**	0.78 (0.33–1.83)
Hispanic x Medicaid	0.85 (0.56–1.29)	0.71 (0.25–1.98)
Asian or Pacific Islander x Medicaid	0.79 (0.39–1.59)	0.18 (0.02–1.92)
Other race x Medicaid	**0.50 (0.27–0.93)**	2.47 (0.43–14.37)
Black race x SUD history	**0.67 (0.46–0.99)**	-
Hispanic x SUD history	0.67 (0.43–1.05)	-
Asian/Pacific Islander x SUD history	0.41 (0.05–3.76)	-
Other race x SUD history	1.03 (0.45–2.36)	-

UDT, urine drug testing; SD, standard deviation; HIV, human immunodeficiency virus;

Ref, reference; SUD, substance use disorder

Bold values are statistically significant at *P* < 0.05

**Table 4:** Negative binomial regression model of factors associated with number of UDTs received

Variable	Beta	*P* value
Age	-0.029	< 0.001
*Race/ethnicity*		
Non-Hispanic White	Ref	Ref
Black	1.117	< 0.001
Hispanic	0.775	< 0.001
Asian/Pacific Islander	0.005	0.981
Other	0.429	0.071
*Insurance type*		
Non-Medicaid	Ref	Ref
Medicaid	0.885	< 0.001
*Prenatal care*		
Gestational age at first visit	-0.001	0.855
*Obstetric history*		
Parity	0.103	< 0.001
*Maternal comorbidities*		
Mental health diagnosis		
No	Ref	
Yes	0.241	< 0.001
STI, HIV, Hepatitis C		
No	Ref	
Yes	0.194	< 0.001
*Maternal substance use history*		
SUD (past history)		
No	Ref	
Yes	1.568	< 0.001
Black race x Medicaid	-0.510	< 0.001
Hispanic x Medicaid	-0.266	0.152
Asian or Pacific Islander x Medicaid	0.044	0.860
Other race x Medicaid	-0.226	0.288
Black race x SUD history	-0.816	< 0.001
Hispanic x SUD history	-0.690	< 0.001
Asian/Pacific Islander x SUD history	-0.368	< 0.001
Other race x SUD history	-0.385	0.013

UDT, urine drug test; SD, standard deviation; HIV, human immunodeficiency virus

Ref, reference; SUD, substance use disorder

Bold values are statistically significant at *P* < 0.05

Primary hypothesis (race) and other SES factors.

Race was associated with testing across multiple outcomes. Black (OR 2.09, 95% CI 1.54–2.84) and individuals of ‘Other’ races (OR 1.64, 95% CI 1.03–2.64) had greater odds of UDTPN compared to non-Hispanic White individuals. Black and Hispanic individuals had a positive relationship with UDTNUM (Beta = 1.12, *p* < 0.001 and Beta = 0.76, *P* < 0.001) compared to non-Hispanic White individuals. No racial differences in UDT were found for UDTDEL. Those insured by Medicaid had greater odds of UDTPN (OR 1.27, 95% CI 1.11–2.49) compared to those with non-Medicaid insurance. There was a positive relationship between being insured by Medicaid and UDTNUM (beta = 0.89, *p* < 0.001). When we examined the interaction terms between race and Medicaid, we found that the association between Black race and Other race and UDTPN was less strong compared to Black and Other race as its own term. Similar results were found for the Black race and SUD interaction term. With the exception of Asian/Pacific Islanders (OR 0.22, 95% CI 0.05–0.95), the association between a positive UDT result and race/ethnicity were not statistically significantly different compared to non-Hispanic White individuals (Table 5).

**Table 5:** Multivariable logistic regression model of factors associated with any positive UDT result based on maternal race and ethnicity^*^

Race	OR (95% CI)	*P* value
Non-Hispanic White	Ref	
Black	0.95 (0.72–1.25)	0.723
Hispanic	0.76 (0.56–1.04)	0.086
Asian/Pacific Islander	0.22 (0.05–0.95)	0.042
Other	0.94 (0.54–1.62)	0.815

UDT, urine drug testing

^*^Model was adjusted for the following covariates: age; gestational age at the first prenatal care visit; payor; presence of mental health condition; composite variable of sexually transmitted infection, hepatitis C, and HIV; prior history of SUD, and parity

### Prenatal Care-Related Factors

We included the gestational age at the first prenatal visit in all models. It was only a factor in UDTDEL where there were greater odds of UDT receipt for every week delay in prenatal care (OR 1.06, 95% CI 1.04–1.07).

### Substance Use-Related Factors

Those who had a past diagnosis of SUD se had greater odds of receiving UDTPN (OR 4.17, 95% CI 2.77–6.27) and UDTDEL (OR 1.48, 95% CI 1.03–2.15). Past diagnosis of SUD also had a positive relationship with UDTNUM (beta = 1.57, *p* < 0.001)

### Maternal Comorbidities

Individuals with a mental health condition had greater odds of UDTPN (OR 1.35, 95% CI 1.16–1.58) and had a positive relationship with UDTNUM (beta = 0.24, *p* < 0.001). Those with an STI, hepatitis C virus, or HIV had greater odds of UDTPN (OR 1.41, 95% CI 1.13–1.75 and a positive relationship with UDTNUM (beta = 0.19, *p* < 0.001).

### Sensitivity Analyses

Similar results were found when we removed 2020 data to account for differences due to the COVID-19 pandemic. When we included only the first live birth for any individuals with more than one live birth during the study period, we found that Black race continued to be associated with UDTPN (OR 2.09, 95% CI 1.53–2.85) but being in the Other race category was no longer significantly associated with UDTPN. We also found similar results for analysis of UDTDEL as the primary analysis with the exception of past diagnosis of SUD, which was no longer associated with UDTDEL in the sensitivity analysis. Similar results to the primary analysis were found for the sensitivity analysis of UDTNUM.

## Discussion

Our study found that race and other markers of SES were associated with UDT during the perinatal period in an urban, academic medical center. Confirming our hypothesis, we found that Black individuals had a 2.1 times greater odds of receiving testing during prenatal care compared to non-Hispanic White patients despite controlling for sociodemographics, markers of prenatal care, and maternal comorbidities. We also found a positive relationship between being of Black or Hispanic race/ethnicity and number of UDTs ordered during perinatal care. Our study did not find race/ethnicity to be a significant predictor of UDTs performed only at the delivery encounter. Black individuals were not more likely to have a positive UDT result compared to non-Hispanic Whites.

Racial disparities in UDT have been examined in prior studies. In 2007, Kunins et al. found that Black individuals had 1.5 times greater odds of testing compared to their White counterparts during pregnancy-related admissions. Kerker et al. (2004) found nearly identical results in an earlier 2004 study that evaluated testing during prenatal care and at delivery. Several recent studies have found evidence of persistent racial disparities in perinatal UDT. Winchester et al. (2022) found evidence of persistent racial disparities in testing at delivery in addition to poor documentation of patient consent for testing. Perlman et al. (2022) found that inpatient pregnant people of color were significantly more likely than White pregnant people to undergo UDT for indications other than prior or current substance use. A recent study by Jarlenski et al. (2023) showed that Black individuals had the greatest probability of UDT at delivery versus White individuals and other racial groups. Chin et al. (2022) found that disparities in testing also apply to other racial/ethnic groups by showing that Native Hawaiians and Pacific Islanders were more likely to receive UDT compared to Whites. Our study adds to the literature on persistent disparities in UDT by showing evidence that disparities in testing also exist during the prenatal care period. Additionally, we examined receipt of multiple tests during obstetrical care and found that similar disparities exist. Unlike prior literature, we did not find race to be a predictor of UDT at the delivery encounter only. However, this analysis may have been limited by a small sample size.

The literature has consistently shown evidence of disparities and selective drug testing by healthcare providers. Experts recommend implementation of policies that uniformly screen all pregnant people (versus selective screening based only on perceived level of risk) for substance use to avoid implicit bias (ACOG, 2017; Terplan & Minkoff, 2017). ACOG recommends that screening be performed verbally using validated tools, such as the 4Ps or National Institute on Drug Abuse Quick Screen. A positive substance use screen should trigger a non-judgmental conversation and referral to treatment in partnership with the patient (ACOG, 2017). Furthermore, it is critical that providers familiarize themselves with local laws surrounding mandatory reporting of prenatal drug use, as 24 states and the District of Columbia consider substance use during pregnancy to be child abuse (McCourt et al., 2022).

Our study has several limitations. First, this was a single center study at one academic health system, potentially limiting the generalizability of our findings. Additionally, the retrospective nature of our study did not allow us to account for every variable that may have influenced an individual receiving UDT. However, every effort was made to include variables that may have influenced providers’ testing decisions to evaluate UDT disparities based on race/ethnicity and SES. We recognize there may be other explanations for these disparities that we were unable to measure. Lastly, this retrospective, single center study allows us to evaluate associations between factors but does not allow us to draw causal conclusions.

## Conclusion

Our findings add to the body of literature demonstrating persistent disparities in substance use testing during the perinatal period leading to racial and SES-related disparities. Future research should investigate health care system-level interventions that aim to mitigate disparities in testing.

## Data Availability

Not Applicable.
